# Assessment of the Malting Process of *Purgatory* Bean and *Solco Dritto* Chickpea Seeds

**DOI:** 10.3390/foods12173187

**Published:** 2023-08-24

**Authors:** Alessio Cimini, Alessandro Poliziani, Lorenzo Morgante, Mauro Moresi

**Affiliations:** Dipartimento per l’Innovazione nei sistemi Biologici, Agroalimentari e Forestali, Università della Tuscia, Via S. C. de Lellis, 01100 Viterbo, Italy; a.cimini@unitus.it (A.C.); alessandro.poliziani@unitus.it (A.P.); lorenzo.morgante.98@gmail.com (L.M.)

**Keywords:** decorticated malted pulses, germination kinetics, in vitro glycemic index, kilning conditions, pulse flour, pulse malting, pulse processing, raffinose and phytate removal, steeping kinetics

## Abstract

This study was aimed at minimizing the anti-nutrient content of the Gradoli *Purgatory* bean (GPB: *Phaseolus vulgaris*) and *Solco Dritto* chickpea (SDC: *Cicer arietinum*) seeds grown in the Latium region of Italy by defining the three steps of their malting process. The water steeping and germination phases were carried out in a 1.0-kg bench-top plant at 18, 25, or 32 °C. By soaking both seeds at 25 °C for 3 h, 95 to 100% of seeds sprouted. There was no need for prolonging their germination process after 72 h, the degradation degree of raffinose in germinated GPBs or SDCs being about 63%, while that of phytic acid being ~32% or 23%, respectively. The steeping and germination kinetics of both seeds were mathematically described via the Peleg and first-order reaction models, respectively. The third step (kilning) was carried out under fluent dry air at 50 °C for 24 h and at 75 °C for 3 h, and yielded cream-colored malted seeds, the cotyledons of which were cyclonically separated from the cuticles and finally milled. Owing to their composition, the decorticated malted pulse flours might be used in the formulation of specific gluten-free food products high in raw proteins and low in phytate, α-oligosaccharides and in vitro glycemic index (GI). Even if their low GI trait was preserved after malting, only the GPB malt flour having a resistant starch-to-total starch ratio ≥ 14% has the potential to be labeled with the health claim for improving postprandial glucose metabolism according to EU Regulation 432/2012.

## 1. Introduction

Legumes are rich in proteins, dietary fibers, and micronutrients and are currently used to prepare several pulse-based food products to replace foods of animal origin in the diet, as well as gluten-free products [1]. Despite their high nutritional profile [2] and sustainable farming [3], their global per capita consumption has stagnated during the last three decades and currently is no more than 21 g/day [4], probably because of their long cooking times, unpleasant flavor, low-digestible proteins, gastrointestinal problems [5], and high content of anti-nutrients (i.e., phytic acid, tannins, enzyme inhibitors, and flatulence-inducing oligosaccharides) [6].

In 2021, the worldwide production of pulses was nearly 89 million metric tons (Mg) [7]. Of this, the production of dry bean accounted for about 27.7 million Mg [8], chickpea production for about 14.3 million Mg [9], and lentil production for about 5.7 million Mg [10]. The largest world producer of dry beans and chickpeas is India with about 6.1 and 9.9 million Mg per year, respectively, while the second producer is Turkey for dry bean production (630 × 10^3^ Mg) or Brazil for chickpea production (2.9 × 10^6^ Mg). In Italy, dry pulse production decreased significantly from about 640,000 Mg in 1960 to 135,000 Mg in 2010 [11]. Even if the trend has started to revert since 2017, the import-to-consumption ratio is still as high as 98% for lentils, 95% for beans, and 59% for chickpeas. 

Among the pulse varieties cultivated in Italy, three (i.e., *Gradoli Purgatory* beans, GPB; *Solco Dritto* chickpeas, SDC; and Onano lentils, OL) are typical of the Latium region [12]. In previous work [13], the lentils of Onano (Viterbo, Italy), which were awarded with the Protected Geographical Indication (PGI) mark by the European Union (PGI-IT-02651; https://www.tmdn.org/giview/gi/EUGI00000017728, accessed 6 August 2023), were submitted to malting and used to prepare fresh egg-pasta using only malted lentil flour. Such a fresh egg pasta was high in raw proteins (24 g/100 g), low in phytate (0.6 g/100 g) and in vitro glycemic index (41%), and basically free of oligosaccharides. 

In this work, the other two pulse varieties were submitted to malting to attempt lowering their anti-nutrient content. In particular, the small, round, and whitish Purgatory bean seeds, similar to Cannellini beans but with a more delicate taste and a thinner skin, have been cultivated in the province of Viterbo (Italy), specifically in the towns of Gradoli, Acquapendente, and Onano, as testified by the traditional menu of the Purgatory Lunch, which has been served at Gradoli in the first day (Ash Wednesday) of the penitential Lenten season since the XVIIth century [14]. The other pulse variety accounted for the so-called *Solco Dritto* (straight furrow) chickpea (SDC) from the furrow tracing performed in the plain beneath the town of Valentano (Italy) on 14 August of every year, its straightness being regarded as a presage of an excellent harvest [15]. These smooth, yellow-beige skinned chickpea seeds have been locally cultivated since the time of Etruscans. 

To minimize the anti-nutrient contents of pulses, various traditional (i.e., dehulling, soaking, boiling, pressure cooking, sprouting, and fermentation) and emerging (i.e., dielectric heating, extrusion, γ-irradiation, ultrasound, and high hydrostatic pressure) processing techniques have been tested with different reduction yields [16]. 

Malting, a conventional process used in the beer industry to obtain malted barley, was successfully applied to reduce the native content of phytic acid and raffinose in two lentil varieties, including the Onano ones [13].

The first aim of this work was to identify the most proper operating conditions of the three phases (i.e., seed steeping, germination, and kilning) of the malting process of Gradoli *Purgatory* beans and *Solco Dritto* chickpeas. The second aim was to describe mathematically the kinetic rate constants of the seed steeping and germination steps. Finally, the third one was to obtain dehulled malted pulse flours low in phytate and α-galactosides as novel ingredients in the formulation of several gluten-free pulse-based food products, such as fresh and dry pastas with low in vitro glycemic index.

## 2. Materials and Methods

### 2.1. Raw Materials

Two varieties of legumes were used in this work. The Gradoli *Purgatory* beans (*Phaseolus vulgaris*) and *Solco Dritto* chickpeas (*Cicer arietinum*) were produced and supplied by *Il Cerqueto* Srl (Acquapendente, Viterbo, Italy). 

### 2.2. Physical Properties of Pulses

Both pulse seeds (as such or malted) were characterized by determining the mean values of the seed weight (m_S_), volume (v_S_), density (ρ_S_), hydration capacity (HC), and swelling capacity (SC) by accounting for 50 seeds [17]. By assuming that each kernel had spherical conformation, it was possible to estimate the mean radius (R_S_) of each seed [18].

### 2.3. Steeping Equipment

The steeping kinetics of each pulse seed was assessed in a bench-top plant, appropriately designed (Figure S1 in the electronic supplement). Each chamber was equipped with two stainless-steel perforated baskets, each one containing up to 1 kg of rehydrated seeds, and a low-temperature immersion circulator type IB-TastemakerCompact10 (Klarstein Chal-Tec GmbH, Berlin, Germany). As soon as the steeping process was completed, both stainless-steel baskets were moved to the germination chamber, where a sensor-type CJMCU-1080 HDC1080 (Texas Instruments, Dallas, TX, USA) was inserted to monitor the relative humidity (RH) and temperature (T) of the air with an accuracy of ±2% RH and ±0.2 °C, respectively. The germination chamber was thermostated at 18, 25, or 32 °C, while a temperature probe-type DS18B20 (Maxim Integrated, San Jose, CA, USA) was used to measure continuously the temperature of the seeds with an accuracy of ±0.5 °C. Water was sprayed inside each germination chamber for 1 min every hour, while the germinating seeds were aerated by manual mixing every 24 h to attempt homogenizing the distribution of water and disrupting the aggregates formed by root sprouting.

Soaking trials were carried out by charging each of the two baskets with about 0.3 kg of dry seeds, which were then submerged with deionized water at 18, 25, or 32 °C. Several soaked kernels were sequentially collected as time increased from 0 to 24 h, rapidly blotted on paper towels, and placed on the weighing plate of a Kern DAB 100-3 thermostatic scale (Kern&Sohn GmbH, Balingen, Germany) to be dried till constant weight at 110 °C for about 20 min.

### 2.4. Seed Germination

During each steeping test, forty seeds were collected at times ranging from 0 to 8 h and laid over an absorbent paper sheet, pre-soaked in 50 mL of deionized water, within a (20 cm × 14.5 cm × 2.5 cm) box. This was sealed and housed in a dark chamber thermostated at the same soaking temperature for 24 or 48 h. All the seeds with a manifest root, independently of its length, were counted and referred to the overall number of seeds accounted for. In this way, it was possible to assess the germination degree after 24 (G_24_) or 48 (G_48_) h, and thus identify the steeping time (t_Sm_) and temperature (T_Sm_), as well as the seed moisture content, associated with the minimum number of non-sprouted seeds. Finally, the seeds moistened at T_Sm_ for as long as t_Sm_ were drained and let germinate up to 24, 48, 72, or 96 h to detect the degradation of phytic acid and α-galactosides using the Phytic Acid and Raffinose/Sucrose/D-Glucose Assay Kits (Megazyme Ltd., Bray, Ireland), respectively.

### 2.5. Germinated Seed Kilning

Both germinated pulses were finally dehydrated at 50 °C for 24 h, and then at 75 °C for 3 h using the Nobel Pro 6 ventilated dryer (Vita 5, Gronsveld, The Netherlands), thus obtaining GPB and SDC malts. By using a portable color-measuring instrument mod. D25-PC2 (Hunterlab, Reston, VA, USA) with a diffuse (0/45°) illuminating viewing geometry, it was possible to assess the color of the split seeds in the CIELAB color space.

### 2.6. Dehulling and Grinding of Malted Pulses

Malted pulse seeds were manually submitted to slight abrasion before being aspirated via a cyclone separator using a suction system. Figure S2a shows the cyclone used, which was designed and produced using a 3D printer. In this way, it was possible to split malted GPB (Figure S2b) or SDC (Figure S2c) seeds into a cotyledon-rich fraction (Figure S2d or Figure S2e) and a cuticle-rich one. An electric stone mill (Mockmill 200, Wolfgang Mock, Otzberg, Germany) was used to convert each cotyledon-rich fraction into a decorticated GPB or SDC malt flour, its fineness being regulated at level 2 out of 10. Total starch (TS) and resistant starch (RS) contents in any malted pulse flour were assayed using the enzymatic kits by Megazyme Ltd. (Bray, Ireland). TS was assessed in raw samples, once dried and ground, while RS was tested in cooked ones.

### 2.7. Cooking of Pulse Seeds as Such or Malted

About 750 g of GPB or SDC seeds were weighted and transferred into a container. After adding tap water at 20 °C using a water-to-seed ratio of 4 g/g [19], the seeds were let to soak at 20 °C for 16 h. Once the soaking water had been drained, the moistened seeds were transferred into a stainless-steel pot containing 3 kg of tap water and let to cook with the lid closed at 98 °C for 60 or 90 min, respectively, using a 2-kW induction-plate hob (INDU, Melchioni Spa, Milan, Italy). About 750 g of decorticated malted GPBs or SDCs were cooked without presoaking at 98 °C for 45 or 60 min, respectively, with the pot initially filled with 3 kg of tap water at room temperature. The hob knob was set at the nominal power of 2 kW till the water started boiling, then it was shifted to 0.4 kW till the end of seed cooking. All the cooked pulses were recovered from the cooking water using a colander, cooled by running tap water for 90 s, and drained.

### 2.8. In Vitro Glycemic Index

In vitro digestion of the cooked pulses as such or malted was carried out using the procedure developed by Zou et al. [20]. All the tests were at least triplicated. By assaying the time course of the concentration of glucose released (C_G_) during the simulated digestion using the *D*-Glucose Assay Procedure K-GLUC 07/11 (Megazyme Ltd., Bray, Ireland), it was possible to plot the so-called digestogram for each cooked sample. The area under the digestogram (AUC) was numerically calculated for a total digestion time of 180 min using the Trapezoidal Rule. The AUC values were referred to the corresponding area estimated for a reference product (i.e., white bread) [21] to calculate the percentage starch hydrolysis index (SHI), this being equal to 100% for the reference white bread. The empirical formula developed by Granfeldt et al. [22] allowed the in vitro glycemic index (GI) to be finally estimated:

GI = 8.198 + 0.862 × SHI(1)

### 2.9. Statistical Analysis of Data

All the tests were carried out at least 3 times to estimate the mean value (µ) and standard deviation (sd) of any parameter assayed. The Tukey Test at a probability level (*p*) of 0.05 was used to assess the statistical significance of any parameter difference. One-way analysis of variance (ANOVA) was also performed using SYSTAT v. 8.0 (SPSS Inc., Chicago, IL, USA, 1998).

## 3. Results and Discussion

### 3.1. Physical Properties

Table 1 shows the main chemico-physical properties of the pulse seeds under study.

As concerning the Gradoli *Purgatory* beans, their crude protein, total starch, phytic acid, and raffinose contents on a dry matter basis were in line with those of the many bean varieties cultivated worldwide [4,23,24,25]. Raw beans are generally classified as very small, small, average, normal, or big size if their seed weight is smaller than 0.2 g, ranges from 0.2 to 0.3 g, from 0.3 to 0.4 g, from 0.4 to 0.5 g, or greater than 0.5 g [25]; GPBs, having an average weight of 0.167 g/seed, are of very small size with an equivalent spherical radius of 0.309 ± 0.005 cm. Their density (ρ_S_) was in line with that of some white bean varieties grown in Tunisia [26]. The swelling capacity (SC) of any seed depends upon its hydration capacity (HC). High HC and SC values favor bean processing (e.g., soaking, germination, decortication, and fermentation) for either extracting active principles or removing anti-nutritional components [27]. Moreover, the higher the hydration capacity, the lower the bean cooking time and hardness will be. HC and SC were found to vary from 0.17 to 0.54 g/seed and from 0.16 to 0.50 cm^3^/seed, respectively, in several genotypes of dry beans cultivated in Turkey [28] and Tunisia [26], probably because of the different seed size, coat thickness, and water absorption characteristics [29]. Common beans may contain from 0.4 to 16.1 g of α-galactosides [30] and from 0.3 to 2.9 g of phytic acid [31] per 100 g of dry mass. The raffinose equivalent content of GPBs was around 5.3 ± 0.3 g/100 g, while the phytic acid content amounted to 1.15 ± 0.12 g/100 g (Table 1).

As concerning the *Solco Dritto* chickpeas, their crude protein, phytic acid, and raffinose contents on a dry matter basis (Table 1) complied with those of several chickpea varieties [32,33]. The seed weight (m_S_), volume (v_S_), hydration (HC), and swelling (SC) capacities were slightly smaller than those of a few Sicilian strains [17], but greater than some Indian cultivars of the Desi and Kabuli types [34]. Its density (1.3 ± 0.1 g/cm^3^) was like that of the Indian chickpeas, but greater than that (1.18 ± 0.15 g/cm^3^) of the Sicilian seeds. Since also in the case of chickpeas, the swelling capacity and hydration capacities were related to the cooking time [35], it is highly likely that the cooking time of SDCs would be intermediate among those of the above Indian and Sicilian varieties. SDCs contained about 3.8 g of raffinose and from 1.15 g of phytic acid per 100 g of dry mass, values quite near to those (4.2 ± 0.7 and 1.21 ± 0.09 g/100 g dm, respectively) of other Kabuli chickpea seeds grown in Egypt [36]. Moreover, the phytic acid content of SDCs was in line with the range of levels (0.3–1.4 g/100 g dm) assayed in several chickpea varieties by Sparvoli et al. [31]. Finally, once split, the GPB and SDC seeds were characterized by the CIELab color coordinates shown in Table 1. The difference in the lightness (L*) between these raw seeds was not statistically significant at *p* = 0.05 (Table 1), but the raw SDCs exhibited greater red-green (a*) and yellow-blue (b*) components than those of raw GPBs. Nevertheless, both split seeds were of a light color quite near to the cream color in the Avery list [37].

### 3.2. Soaking Kinetics of Dry Legumes

**Figure 1:**
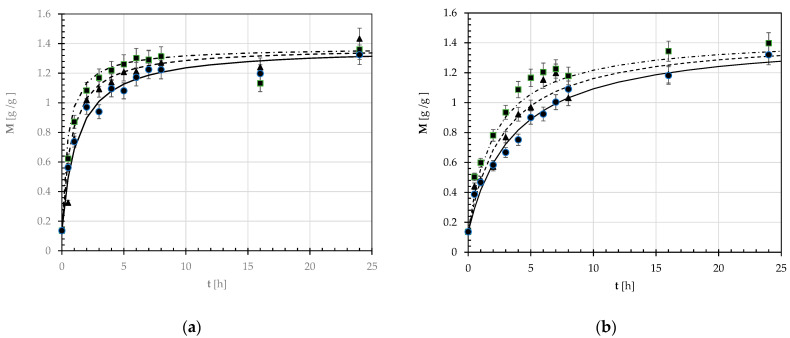
Time course of the experimental moisture weight ratio (M) during the steeping of Gradoli *Purgatory* beans (**a**) and *Solco Dritto* chickpeas (**b**) at different temperatures (●: ―, 18 °C; ▲: - - -, 25 °C; ■, ―. ―, 32 °C). The continuous, broken, and dash-dotted line curves were plotted using the Peleg model (Equation (2)) and the constants k_1_ and k_2_ calculated as reported in the text.

Figure 1 shows the time course of the moisture ratio (M) at the soaking temperatures of 18, 25, or 32 °C for both the pulse varieties under study. Table S1 in the electronic supplement shows the mean values (μ) and standard deviations (sd) of the experimental moisture weight fraction (x_W_) against soaking time. All isotherms were characterized by an initial quick increase for t varying from 0 to about 3 or 5 h in the case of GPB or SDC seeds, respectively. After that, M exhibited a slower growth up to the equilibrium moisture ratio.

The time course of the experimental moisture ratio (M) was described using the empirical model developed by Peleg [38]:

(2) $$M\left(t\right)=M_{0}+\frac{t}{k_{1}+{\;k}_{2}t}$$

where t is the soaking time, M_0_ is the initial moisture ratio, while k_1_ and k_2_ are the Peleg rate and capacity constants.

The mean values and standard deviations of both Peleg constants were determined using the least squares method upon linearization of Equation (2):

(3) $$\frac{t}{M\left(t\right)\;-\;M_{0}}=k_{1}+{\;k}_{2}\;t$$

as shown in Table 2 together with the corresponding coefficient of determination (r^2^).

**Figure 2:**
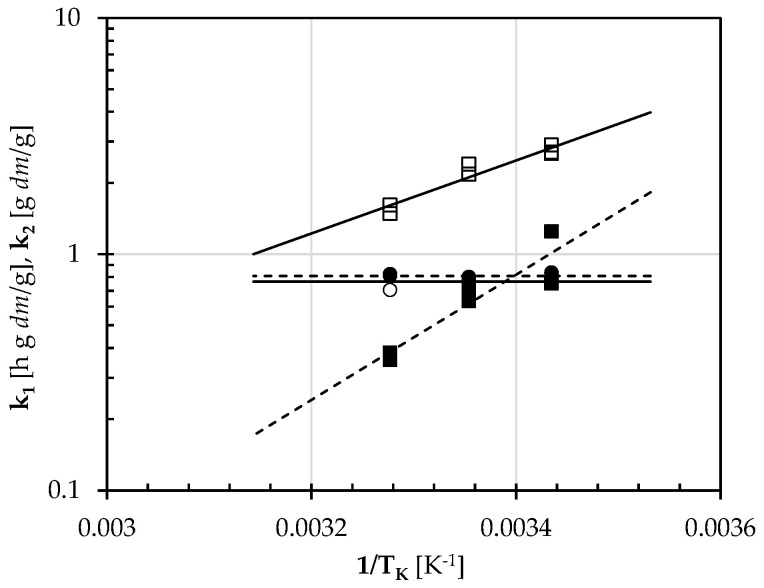
Peleg constants k_1_ and k_2_ of the soaking kinetics of Gradoli Purgatory beans (closed symbols and broken lines) and *Solco Dritto* chickpeas (open symbols and continuous lines): experimental and calculated k_1_ (■, ☐) and k_2_ (●, ○) values as a function of the reciprocal of the absolute soaking temperature (T_K_). The broken and continuous lines fitting the k_1_ values were plotted using Equation (4) with the parameters listed in Table 2, while the horizontal ones were plotted by averaging the k_2_ values shown in Table 2.

Since the reciprocal of k_1_ coincides with the initial water uptake rate (R_W0_), its dependence on the absolute soaking temperature (T_K_) was described using the following Arrhenius-type relationship:

(4) $$\frac{1}{k_{1}}=A\;\exp(-\frac{E_{a}}{R\;T_{K}})$$

where E_a_ is the activation energy, A is the pre-exponential nonthermal factor, and R (=8.31 J K^−1^ mol^−1^) is the universal gas constant. The semilogarithmic plot shown in Figure 2 confirmed this assumption for both the pulses examined. On the contrary, the estimated k_2_ values appeared to be about constant ($\bar{k_{2}}$) and temperature-independent, as also observed by other authors [39,40,41]. Table 2 also lists the least-squares estimated values of *A* and (*E_a_/R*), as well as$\;\bar{k_{2}}.$

Finally, Figure 1 compares the experimental and calculated M values at the three temperatures examined for both pulses. For all the hydration isotherms examined, the average experimental error among the experimental and calculated M values was around 10 or 8% for the GPB or SDC seeds, respectively.

For both the legumes under study, the independent parameters (*A* and *E_a_/R*) were found to be statistically different at *p* = 0.05, while the Peleg capacity constant (k_2_) was practically constant (0.78 ± 0.04 g dm/g) (Table 2).

As t→∞, the moisture ratio M approaches the equilibrium moisture content (M_e_):

(5) $$M_{e}=M_{0}+\frac{1}{k_{2}}$$

The estimated value of the equilibrium moisture weight ratio (M_e_) or fraction (x_We_) for both legumes was approximately equal to 1.42 g/g dm or 58.6% *w*/*w*, respectively, in agreement with the values extrapolated from the data in Table S1.

### 3.3. Pulse Germinability

**Figure 3:**
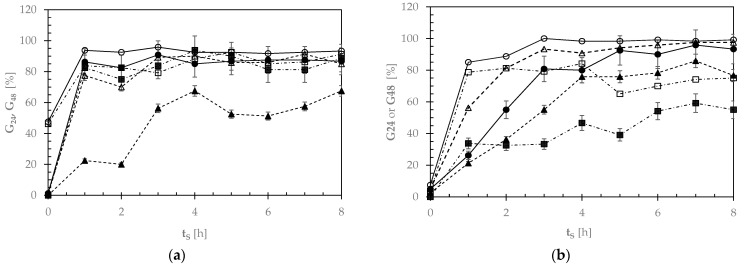
Average percentage of (**a**) Gradoli Purgatory beans and (**b**) *Solco Dritto* chickpeas germinated after 24 (G24: closed symbols) or 48 (G48: open symbols) h, once they had been presoaked at different temperatures (18 °C: ▲, △, - - -; 25 °C: ●, ○, ―; 32 °C: ■, ☐, ―. ―) for different steeping times (t_S_).

During the pulse seed soaking at 18, 25, or 32 °C, forty seeds were collected at times ranging from 0 to 8 h and let germinate at the same steeping temperature up to 24 or 48 h. Figure S3 shows the sealed boxes used to determine the soaking temperature and time associated with the maximum number of sprouted seeds. Table S2 reports the average number of GPBs and SDCs, exhibiting an evident root despite its longer or shorter length. By plotting the degree of germinability (G), that is the percentage of sprouted seeds out of 100 seeds, against the steeping time (t_S_) (see Figure 3), it was noted that GPB seeds tended to sprout quicker than the SDC ones. However, after 48-h germination at 25 °C, 96 ± 4% of all the GPB seeds, but almost 100% of the SDC seeds sprouted after a presoaking time of 3 h. In such soaking conditions, which minimized the degree of inhomogeneity in germinating seeds, the average moisture content of the GPB or SDC seeds was equal to 52 ± 4 or 43 ± 3% (*w*/*w*), respectively (Table S1).

### 3.4. Pulse Germination

Once the GPB and SDC seeds had been soaked at the above optimal conditions (T_sm_ = 25 °C, t_sm_ = 3 h), and the steeping water drained out, both rehydrated seeds were let to germinate at 25 °C up to 96 h.

Seed germination involves three steps, namely water imbibition, reactivation of metabolism, and radicle protrusion [42]. As seeds imbibe water, some physiological and biochemical processes (i.e., hydrolysis, macromolecules biosynthesis, respiration, subcellular structures, and cell elongation) are reactivated. These result in the hydrolysis of stored starch, polyphosphate, and other storage materials into simple forms. For instance, germinating seeds use sugars and other molecules as a substrate for respiration. The greatest storage form of total phosphorus (about 50–80%) in legumes is phytic acid (C_6_H_18_O_24_P_6_). This anti-nutrient may form complexes with proteins, and chelate some cations (i.e., Fe, Ca, K, Mn, Mg, Zn). The resulting mixed salts, such as phytin or phytate, in germinating seeds are hydrolyzed by an acid phosphatase enzyme (phytase), thus freeing phosphate, cations, and inositol easily utilizable by the seedlings [42].

**Figure 4:**
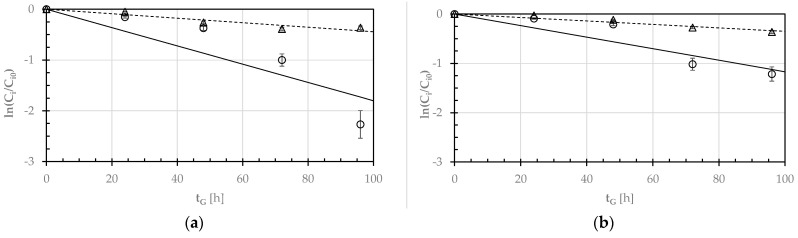
Semi-logarithmic diagram of the ratio (C_i_/C_i0_) for the raffinose (R: ○. ―) and phytic acid (PA: △, - - - -) concentrations during the germination of GPBs (**a**) and SDCs (**b**) at 25 °C as a function of the germination time (t_G_). The continuous and broken lines were plotted using the first-order kinetic model (Equation (6)) and the kinetic constant rates reported in Table 3.

Since the plot of the natural logarithm of the ratio between the current (C_i_) and initial (C_i0_) concentrations of the i-th anti-nutrient against the germination time (t_G_) exhibited a linear negative trend for both the pulse seeds examined (Figure 4), the degradation kinetics of raffinose (R) or phytic acid (PA) were described as a first order reaction:

(6) $$\frac{d{\;C}_{i}}{dt}={-\;k}_{i}\;C_{i}$$

where k_i_ is the degradation kinetic rate constant of the i-th anti-nutrient. By separating the independent variables and integrating, the following was obtained:

(7) $$C_{i}=C_{i0\;}e^{{-k}_{i}t}\;for\;t\geq 0$$

where C_i0_ is the initial concentration of the i-th component.

Table 3 shows the least-squares estimates of k_i_ for both the pulses of concern.

It can be noted that the degradation rate constant of raffinose during the germination of GPBs was one and a half greater than that relative to the germinating SDCs, while the degradation rate constant of phytic acid for both seeds was not statistically different at *p* < 0.05.

**Figure 5:**
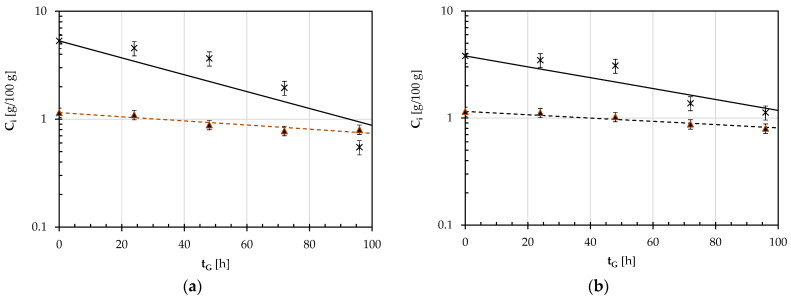
Germination of GPBs (**a**) and SDCs (**b**) at 25 °C: Concentration (C_i_) of raffinose (R: ∗, —) or phytic acid (PA: ▲, - - -) against the germination time (t_G_). The continuous and broken lines were plotted using the first-order kinetic model (Equation (7)) and the kinetic constant rates reported in Table 3.

Finally, Figure 5 shows the evolution of the concentration of raffinose and phytic acid during the germination of GPBs and SDCs at 25 °C. The lines plotted were calculated using Equation (7) with the kinetic constant rates shown in Table 3. It can be noted as quite a good reconstruction of the experimental profiles.

In summary, there was no need for extending the seed germination process at 25 °C up to 96 h (Table S3). After 72-th, the degradation degree of raffinose or phytic acid in the germinating GPBs and SDCs was about 62% or 32%, and 63% or 23%, respectively. These phytate degradation degrees were smaller than those reported for a 96-h germination of Kabuli-type chickpeas (73%) and kidney bean from Ethiopia (79–96%) at 25 °C [43], as well as those measured in germinated black (53%) and white (45%) [44]. As concerning the only soaking in water of common beans and chickpeas, the reduction degree of raffinose or phytate was found to range from 22 to 97% or from 0.2 to 35%, respectively [16].

### 3.5. Malted Pulse Flour Production and Characterization

Once dehydrated firstly at 50 °C for 24 h and then at 75 °C for 3 h, the germinated pulse seeds gave rise to malted seeds having a moisture content of about 10% (*w*/*w*).

By using the cyclone shown in Figure S2a, almost all the cuticle fragments were separated from the cotyledons of both malted pulses. The cotyledon-rich fraction recovered approximately represented 85% or 86% of the input malted GPBs or SDCs, respectively.

As shown in Table 1, there was a general reduction in the physical properties of the decorticated malted pulses with respect to raw pulse ones. Moreover, the content of raffinose or phytate in malted GPBs or SDCs was reduced to about 37% or 68% of the original one, respectively.

Table 1 also shows the CIELab coordinates of the split malted seeds. The difference in their lightness L* was not statistically significant at *p* = 0.05, but the malted SDCs exhibited greater red-green (a*) and yellow-blue (b*) components than malted GPBs.

The difference in the CIELab coordinates (L*, a*, b*) between the raw and malted GPBs was not statistically significant, while the malted SDCs had greater lightness and a smaller red-green component (a*) than the raw counterpart, but the same yellow-blue component (b*). Since chickpea is a rich source of carotenoids, such as xanthophyll (9.0–19.7 mg/100 g), canthoxanthine (21.0–67.9 mg/100 g), and β-carotene (166–431 μg/100 g) [45], the significant increase in the lightness L* of malted SDCs may be highly likely attributed to the oxidative reactions, especially *cis-trans* isomerization of carotenoids, that occur during air kilning, these being intensified by higher temperature and lower relative humidity drying conditions [46]. Altogether, both malted seeds displayed a light color of the cream type in the Avery list [37].

Upon grinding for two or three cycles, it was possible to obtain a dehulled GPB or SDC malt flour, their raw protein, total starch, resistant starch, raffinose, and phytic acid contents coinciding with those of decorticated malted pulse seeds (Table 1).

Both these flours were characterized by almost the same raw protein (~23.5 g/100 g dm), raffinose (~1.8 g/100 g dm), and phytic acid (~0.78 g/100 g dm) contents, but quite different total and resistant starch concentrations. Of these, owing to its low total starch content (~35 g/100 g dm) and high resistant starch-to-total starch ratio (~0.63 g/g), the dehulled GPB malt flour might represent a valuable ingredient for the formulation of functional foods having a resistant starch level ≥ 14% of their TS content, this allowing their labelling with a specific health claim regarding the physiological effect of improved postprandial glucose metabolism according to EU Regulation 432/2012 [47].

### 3.6. In Vitro Glycemic Index of Pulse Seeds

**Figure 6:**
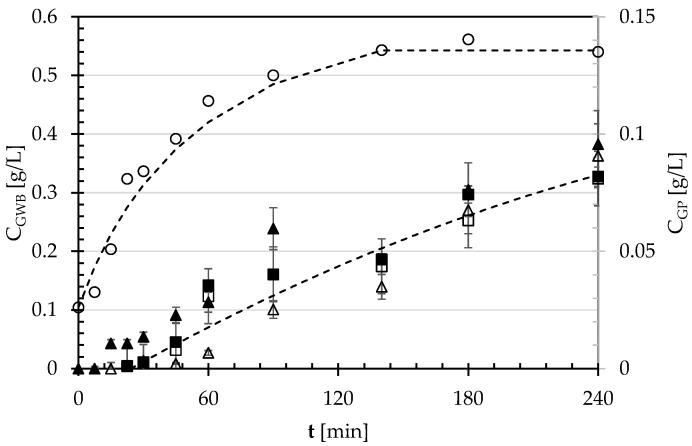
Glucose concentration freed by the simulated in vitro digestion of white bread (C_GWB_: ○) or cooked pulses (C_GP_) versus time (t): GPBs as such (☐) or malted (■); SDCs as such (△) or malted (▲).

To describe the simulated digestion kinetics of the cooked pulses as such or malted, the average concentration (C_G_) of glucose freed by the enzymatic treatments was plotted against the incubation time (t), as shown in Figure 6.

Figure 6 shows that white bread exhibits a glycemic index significantly greater than the pulses, as also confirmed by the numerical calculation of the areas under each digestogram (AUC) up to an overall incubation time of 180 min (cfr. Table 4). It can be noted that AUC reduced from about 81 g min/L in the case of white bread to as low as 4.2 or 4.9 g min/L for the cooked SDC or GPBs seeds as such, the difference between these AUC values being not statistically significant at *p* = 0.05. For the malted GPB and SDC seeds, the AUC values were slightly higher (6.0 or 7.6 g min/L), but even their difference was statistically negligible at *p* = 0.05. Thus, by using Equation (1), the estimated in vitro glycemic index (GI) of both GPBs and SDCs as such or malted was equal to ~13 or 15%, respectively. Since such malted flours had a resistant starch content not statistically different from that of their native forms (Table 1), the in vitro tests shown in Figure 6 suggested that the malting process did not significantly change the GI response of the pulses.

Probably because of the different methods of preparation, processing, and heat application, the glycemic indexes reported in the literature [21] exhibit a wide variation from 18 ± 2 to 99 ± 11% for common beans, and from 14 ± 3 to 96 ± 21% for chickpeas when using white bread as a reference.

According to Foster-Powell et al. [48] and Atkinson et al. [49], foods can be classified into three categories: low (≤55), medium (55–69), and high (≥70) GI foods. Thus, the malting process did not affect the in vitro GI of the pulses examined here.

## 4. Conclusions

All the operating conditions of the three steps of the malting production process of two decorticated malted Gradoli *Purgatory* bean and *Solco Dritto* chickpea flours were defined together with the mathematical modelling of their seed steeping and germination processes. A three-h water steeping followed by a 72-h germination at 25 °C was sufficient to reduce the raffinose or phytic acid content by about 38% or 68–77% of the corresponding native content, respectively.

The subsequent kilning at 50 °C for 24 h and at 75 °C for 3 h gave rise to malted pulse seeds having almost the same cream color. The decorticated malted Gradoli *Purgatory* bean and *Solco Dritto* chickpea flours were characterized by quite different total and resistant starch concentrations, but almost the same raw protein (~23.5 g/100 g dm), raffinose (~1.8 g/100 g dm), and phytic acid (~0.78 g/100 g dm) contents, their low GI trait being preserved after malting. Thus, these flours might be regarded as valuable ingredients for designing several gluten-free food product formulations low in fats, α-oligosaccharide, and phytate specific for celiac, diabetic, and hyperlipidemic patients. However, just those prepared with the dehulled GPB malt flour (having a resistant starch level by far greater than 14% of its total starch content) might be labelled with the health claim of improved postprandial glucose metabolism according to EU Regulation 432/2012. Further work is needed to test the technical feasibility and sensory properties of such novel formulations.

## Figures and Tables

**Table 1:** Main chemico-physical properties and CIELab coordinates (L*, a*, b*) of Gradoli *Purgatory* bean (GPB) and *Solco Dritto* chickpea (SDC) seeds as such or malted (M).

Parameter	GPB	MGPB	SDC	MSDC	Unit
Raw protein	22.7 ± 1.7 ^a^	23.4 ± 2.1 ^a^	22.3 ± 1.7 ^a^	23.6 ± 1.9 ^a^	g/100 g dm
Total Starch (TS)	33.81 ± 1.66 ^b^	34.96 ± 0.19 ^b^	46.8 ± 0.6 ^a^	45.2 ± 2.0 ^a^	g/100 g dm
Resistant Starch (RS)	23.59 ± 0.34 ^a^	22.01 ± 1.82 ^a^	1.77 ± 0.22 ^b^	1.19 ± 0.43 ^b^	g/100 g dm
Phytic Acid (PA)	1.15 ± 0.12 ^a^	0.78 ± 0.13 ^b^	1.15 ± 0.12 ^a^	0.79 ± 0.09 ^b^	g/100 g dm
Raffinose (R)	5.31 ± 0.28 ^a^	1.95 ± 0.20 ^c^	3.80 ± 0.15 ^b^	1.65 ± 0.11 ^c^	g/100 g dm
Seed weight (m_S_)	0.167 ± 0.003 ^c^	0.133 ± 0.002 ^d^	0.302 ± 0.010 ^a^	0.219 ± 0.002 ^b^	g/seed
Seed volume (v_S_)	0.123 ± 0.006 ^c^	0.103 ± 0.000 ^d^	0.233 ± 0.012 ^a^	0.191 ± 0.000 ^b^	cm^3^/seed
Mean seed radius (R_S_)	0.309 ± 0.005 ^c^	0.290 ± 0.000 ^d^	0.382 ± 0.006 ^a^	0.358 ± 0.000 ^b^	cm/seed
Seed density (ρ_S_)	1.35 ± 0.03 ^a^	1.30 ± 0.002 ^b^	1.30 ± 0.09 ^a,b^	1.14 ± 0.01 ^c^	g cm^3^
Hydration capacity (HC)	0.149 ± 0.006 ^c^	0.132 ± 0.003 ^d^	0.284 ± 0.006 ^a^	0.182 ± 0.010 ^b^	g/seed
Swelling capacity (SC)	0.313 ± 0.012 ^c^	0.368 ± 0.007 ^b^	0.503 ± 0.015 ^a^	0.369 ± 0.012 ^b^	cm^3^/seed
L*	71.0 ± 1.7 ^b,c^	73.3 ± 1.5 ^a,b^	69.5 ± 1.6 ^c^	75.1 ± 1.8 ^a^	-
a*	0.6 ± 0.5 ^c^	0.01 ± 0.61 ^c^	3.7 ± 0.5 ^a^	2.3 ± 0.6 ^b^	-
b*	15.6 ± 1.7 ^b^	19.0 ± 1.9 ^b^	27.0 ± 2.3 ^a^	27.0 ± 1.3 ^a^	-

In each row, values with the same letter have no significant difference at *p* < 0.05.

**Table 2:** Mean values and standard deviations (μ ± sd) of Peleg constants k_1_ and k_2_ at different steeping temperatures (T) for Gradoli *Purgatory* beans and *Solco Dritto* chickpeas and empirical parameters [*Ea*/*R*; *ln(A)*] of the Arrhenius-type relationship (Equation (4)) and mean value $\left(\bar{k_{2}}\right)$.

Legume Variety Parameter	Gradoli *Purgatory* Beans			*Solco Dritto* Chickpeas			Unit
T	18	25	32	18	25	32	[°C]
k_1_	0.92 ± 0.18	1.16 ± 0.29	0.36 ± 0.13	2.76 ± 0.26	2.23 ± 0.35	1.49 ± 0.14	[h g dm/g]
k_2_	0.83 ± 0.02	0.75 ± 0.03	0.81 ± 0.01	0.75 ± 0.03	0.73 ± 0.04	0.71 ± 0.03	[g dm/g]
r^2^	0.996	0.986	0.998	0.988	0.979	0.990	[-]
*E_a_/R*	6099 ± 1278 ^a^			3552 ± 441 ^b^			[K]
*ln(A)*	−20.94 ± 4.29 ^a^			−11.16 ± 1.48 ^b^			[-]
r^2^	0.85			0.93			[-]
$\bar{k_{2}}$	0.81 ± 0.02 ^a^			0.76 ± 0.04 ^a^			[g dm/g]

In each row, values with the same letter have no significant difference at *p* < 0.05.

**Table 3:** Mean values and standard deviations (μ ± sd) of the degradation kinetic rate constant k_i_ of each i-th component (R, PA) during the germination of Gradoli Purgatory beans and *Solco Dritto* chickpeas together with the corresponding coefficients of determination (r^2^).

Parameter	k_i_ [h^−1^]	r^2^	k_i_ [h^−1^]	r^2^
**Component i**	Gradoli *Purgatory* beans		*Solco Dritto* chickpeas	
Raffinose	−0.018 ± 0.003 ^a^	0.89	−0.012 ± 0.002 ^b^	0.92
Phytic acid	−0.0044 ± 0.0005 ^a^	0.96	−0.0035 ± 0.0003 ^a^	0.97

In each row, values with the same letter have no significant difference at *p* < 0.05.

**Table 4:** Estimation of the areas (AUC) enclosed by the digestograms of white bread, Gradoli *Purgatory* beans as such (GPB) or malted (MGPB), and *Solco Dritto* chickpeas as such (SDC) or malted (MSDC) for a digestion time of 180 min using the Trapezoidal Rule, starch hydrolysis index (SHI), and in vitro glycemic index (GI) via Equation (1), and their classification according to the so-called GI chart.

Food Product	AUC [g min/L]	SHI [%]	GI [%]	GI Chart
White bread	81.2 ± 0.4 ^a^	100.0 ± 0.5 ^a^	94.4 ± 0.4 ^a^	High
GPB as such	4.9 ± 0.2 ^c^	6.0 ± 0.3 ^c^	13.4 ± 0.2 ^c^	Low
MGPB	6.0 ± 0.8 ^b,c^	7.4 ± 1.0 ^b,c^	14.6 ± 0.9 ^b,c^	Low
SDC as such	4.2 ± 1.3 ^c^	5.1 ± 1.5 ^c^	12.6 ± 1.3 ^c^	Low
MSDC	7.6 ± 1.1 ^b^	9.3 ± 1.3 ^b^	16.2 ± 1.1 ^b^	Low

In each column, values with the same letter have no significant difference at *p* < 0.05.

## Data Availability

The data used to support the findings of this study can be made available by the corresponding author upon request.

## Supplementary Materials

The following supporting information can be downloaded at: https://www.mdpi.com/article/10.3390/foods12173187/s1, Figure S1: Views of the experimental bench-top soaking chamber used; Figure S2: Picture of the laboratory-scale cyclone used to recover cotyledon-rich fractions; Figure S3: Pictures of the GPBs and SDCs during their germination in sealed boxes at different times; Table S1: Time course of the moisture weight fraction of GPBs and SDCs at different temperatures; Table S2: Average number of germinated GPBs and SDCs at different germination temperatures and times; Table S3: Effect of the germination time on the raffinose and phytic acid contents of GPBs and SDCs at 25 °C.
