# Supplementary material for: Assessment of the Malting Process of Purgatory Bean and Solco Dritto Chickpea Seeds
Source: Foods. 2023 Aug 24;12(17):3187. doi: 10.3390/foods12173187 (PMC10486599; doi:10.3390/foods12173187)
Supplement: Supplementary file 1 [file foods-12-03187-s001.zip › foods-2550163-supplementary.pdf]

Assessment of the malting process of *Purgatory* bean and *Solco Dritto* chickpea seeds

Alessio Cimini, Alessandro Poliziani, Lorenzo Morgante, and Mauro Moresi*

Dipartimento per l'Innovazione nei sistemi Biologici, Agroalimentari e Forestali, Università della Tuscia, Via S. C. de Lellis, 01100 Viterbo, Italy

Table S1

Mean values (μ) and standard deviations (sd) of moisture weight fraction (x_W) and estimated moisture weight ratio (M) of Gradoli Purgatory beans and *Solco Dritto* chickpeas at different steeping times (t_S) and temperature (T_S).

**Lentil variety**	**Gradoli Purgatory Beans**						**Solco Dritto Chickpeas**					
**T_S [°C]**	18		25		32		18		25		32	
**t_S**	**x_W**	M	**x_W**	M	**x_W**	M	**x_W**	M	**x_W**	M	**x_W**	M
[h]	[*]	[§]	[*]	[§]	[*]	[§]	[*]	[§]	[*]	[§]	[*]	[§]
0	12.0±0.0	0.136	12.0±0.0	0.136	12.0±0.0	0.136	12.0±0.0	0.136	12±0.00	0.136	12.0±0.0	0.136
0.5	36.0±1.0	0.563	24.5±1.0	0.325	38.4±0.9	0.623	27.9±1.0	0.388	30.6±0.5	0.440	33.5±1.1	0.503
1	42.5±2.9	0.738	43.0±3.7	0.755	46.6±1.9	0.872	31.8±0.6	0.467	32.6±3.4	0.483	37.4±1.0	0.598
2	49.3±2.2	0.971	50.5±2.8	1.019	52.0±2.1	1.083	36.8±2.0	0.584	36.4±4.1	0.572	43.9±4.1	0.781
3	48.4±0.9	0.940	52.2±3.6	1.093	53.9±1.2	1.170	40.0±2.3	0.669	43.5±3.4	0.770	48.3±2.9	0.935
4	52.3±0.9	1.095	53.2±1.2	1.139	54.9±1.4	1.220	42.9±1.9	0.753	48.0±3.0	0.923	52.1±2.5	1.088
5	51.9±2.6	1.081	54.7±1.4	1.208	55.8±0.8	1.261	47.4±1.4	0.901	49.0±5.0	0.969	53.8±1.4	1.166
6	54.0±1.2	1.173	54.8±0.8	1.212	56.6±0.6	1.302	48.0±0.5	0.924	53.6±2.6	1.153	54.6±0.7	1.205
7	55.0±1.2	1.224	56.3±0.6	1.287	56.4±0.7	1.291	50.1±1.2	1.004	54.5±0.7	1.198	55.1±0.7	1.226
8	55.0±0.8	1.223	56.0±0.9	1.271	56.8±0.9	1.313	52.2±0.6	1.091	50.8±6.4	1.031	54.1±3.1	1.179
16	54.5±0.1	1.197	55.4±0.5	1.241	53.1±4.5	1.132	54.2±1.8	1.183	54.3±0.5	1.190	57.3±1.3	1.344
24	57.0±1.1	1.325	58.9±1.0	1.433	57.6±1.0	1.361	56.9±1.4	1.320	58.3±0.9	1.398	58.3±0.8	1.397

* % w/w

§ g/g *dm*

Table S2: Mean values and standard deviations (μ ± sd) of the average number of germinated Gradoli Purgatory beans and *Solco Dritto* chickpeas previously soaked in water for different steeping times (t_S) and let germinated at different temperatures (T_G) and germination times (t_G).

**t_G [h]**	**24**			**48**		
**T_G [°C]**	**18**	**25**	**32**	**18**	**25**	**32**
**t_S [h]**	***Gradoli Purgatory beans***					
0	0.0 ± 0.0	0.5 ± 0.7	0.0 ± 0.0	0.5 ± 0.0	19.0 ± 12.7	18.5 ± 2.1
1	9.0 ± 7.1	34.5 ± 4.9	33.0 ± 2.8	31.0 ± 5.7	37.5 ± 2.1	33.5 ± 0.7
2	8.0 ± 1.4	33.0 ± 5.7	30.0 ± 8.5	28.0 ± 5.7	37.0 ± 1.4	33.0 ± 7.1
3	22.5 ± 9.2	36.3 ± 1.5	33.5 ± 4.9	35.7 ± 4.0	38.3 ± 1.5	31.7 ± 9.2
4	27.0 ± 5.7	34.0 ± 4.6	37.5 ± 2.1	36.0 ± 2.0	37.0 ± 4.4	35.3 ± 4.0
5	21.0 ± 1.4	34.7 ± 3.5	36.0 ± 0.0	34.3 5.5	37.0 ± 3.0	37.0 1.4
6	20.5 ± 0.7	35.0 ± 3.6	32.5 ± 2.1	34.3 ± 1.2	36.7 ± 2.5	34.0 ± 0.0
7	23.0 ± 1.4	35.0 ± 4.6	32.5 ± 3.5	36.5 ± 0.7	37.0 ± 3.5	34.5 ± 2.1
8	27.0 ± 1.4	34.7 ± 4.2	35.5 ± 2.1	34.0 ± 1.4	37.3 ± 1.5	36.5 ± 2.1
**t_S [h]**	***Solco Dritto chickpeas***					
0	1.0 ± 1.4	2.0 ± 2.8	0.0 ± 0.0	2.0 ± 0.0	3.0 ± 4.2	1.5 ± 0.7
1	8.5 ± 0.7	10.5 ± 3.5	13.5 ± 0.7	22.5 ± 9.2	34.0 ± 7.1	31.5 ± 0.7
2	14.5 ± 0.7	22.0 ± 2.8	13.0 ± 7.1	32.5 ± 6.4	35.5 ± 3.5	32.5 ± 4.9
3	22.0 ± 4.6	32.3 ± 3.5	13.3 ± 5.5	37.3 ± 1.2	40.0 ± 0.0	31.7 ± 3.2
4	30.3 ± 0.6	32.0 ± 4.4	18.7 ± 9.0	36.3 ± 4.6	39.3 ± 0.6	33.7 ± 5.0
5	30.3 ± 3.1	37.0 ± 2.0	15.7 ± 6.0	37.7 ± 0.6	39.3 ± 0.6	26.0 ± 4.6
6	31.3 ± 3.1	36.0 ± 2.6	29.5 ± 13.4	38.3 ± 2.1	39.7 ± 0.6	35.5 ± 0.7
7	34.3 ± 3.8	38.3 ± 0.6	31.0 ± 9.9	39.0 ± 1.0	39.3 ± 0.6	33.0 ± 1.4
8	30.7 ± 0.6	37.3 ± 1.2	27.0 ± 2.8	39.0 ± 0.0	39.7 ± 0.6	33.5 ± 2.1

Table S3

Germination of Gradoli Purgatory bean and *Solco Dritto* chickpeas at 25 °C: effect of the germination time (t_G) on the mean values and standard deviation (μ ± sd) of their raffinose (R) and phytic acid (PA) contents.

**Lentil variety**	**Gradoli Purgatory beans**		***Solco Dritto* chickpeas**	
**t_G**	**R**	**PA**	**R**	**PA**
[h]	[g/100 g dm]			
0	5.3 ± 0.3	1.15 ± 0.12	3.8 ± 0.1	1.15 ± 0.12
24	4.6 ± 0.5	1.10 ± 0.11	3.5 ± 0.4	1.12 ± 0.11
48	3.7 ± 0.3	0.89 ± 0.01	3.1 ± 0.2	1.03 ± 0.01
72	2.0 ± 0.2	0.78 ± 0.13	1.4 ± 0.3	0.88 ± 0.13
96	0.5 ± 0.1	0.80 ± 0.06	1.1 ± 0.1	0.80 ± 0.06

Figure S1

External (**a**) and internal (**b**) views of the bench-top soaking chamber used to rehydrate the pulse seeds under study.

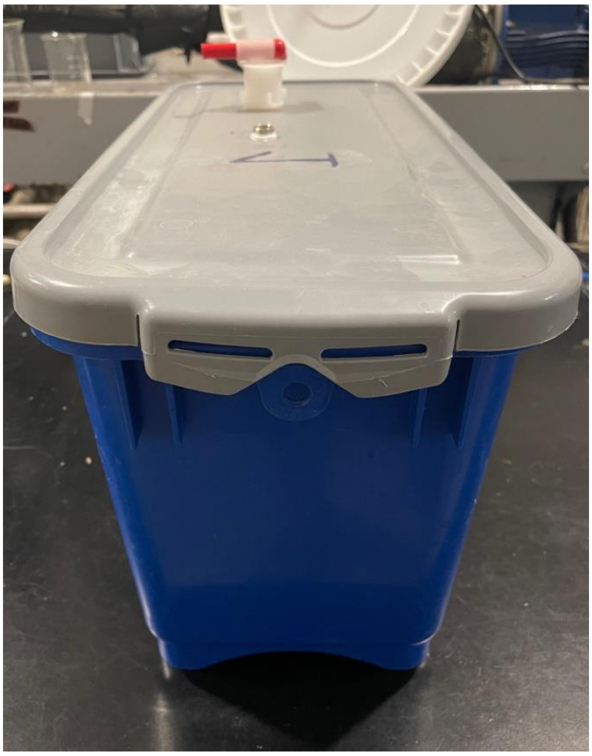

(a)

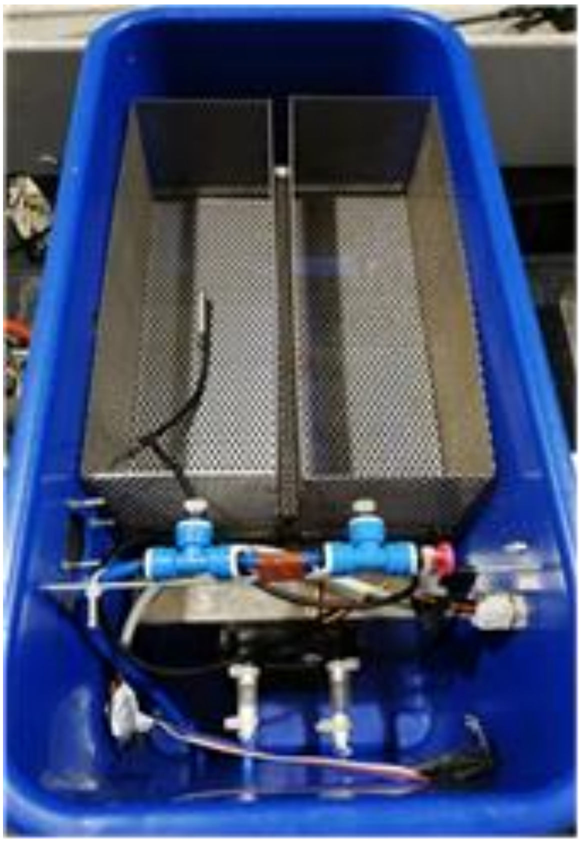

(b)

Figure S2

Picture of the laboratory-scale cyclone (**a**) used to treat the split malted GPB (**b**) or SDC (**c**) seeds and recover a cotyledon-rich fraction of them (**d** or **e**).

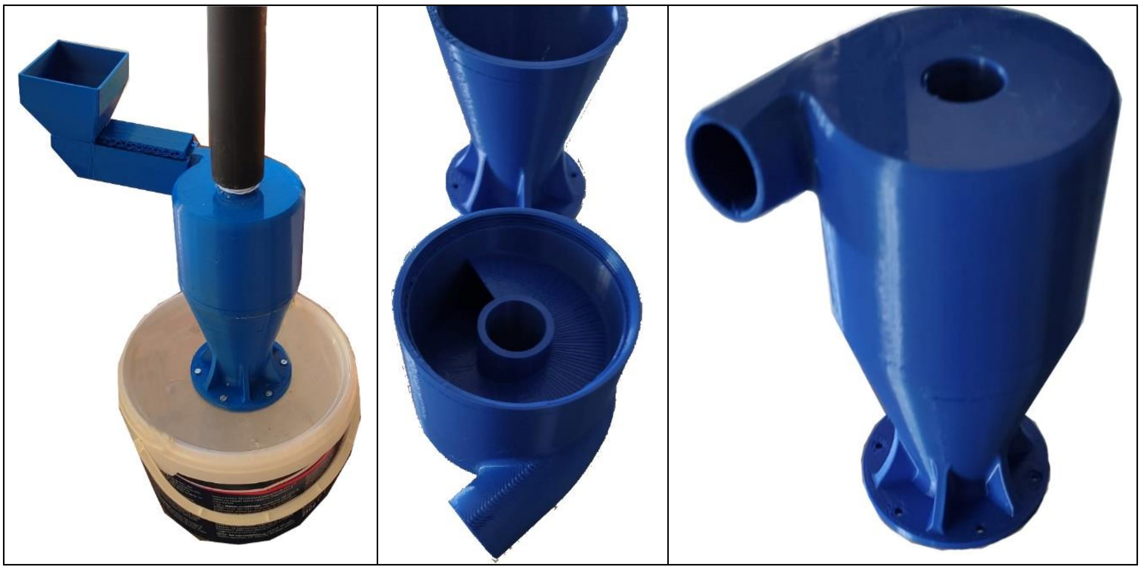

(a)

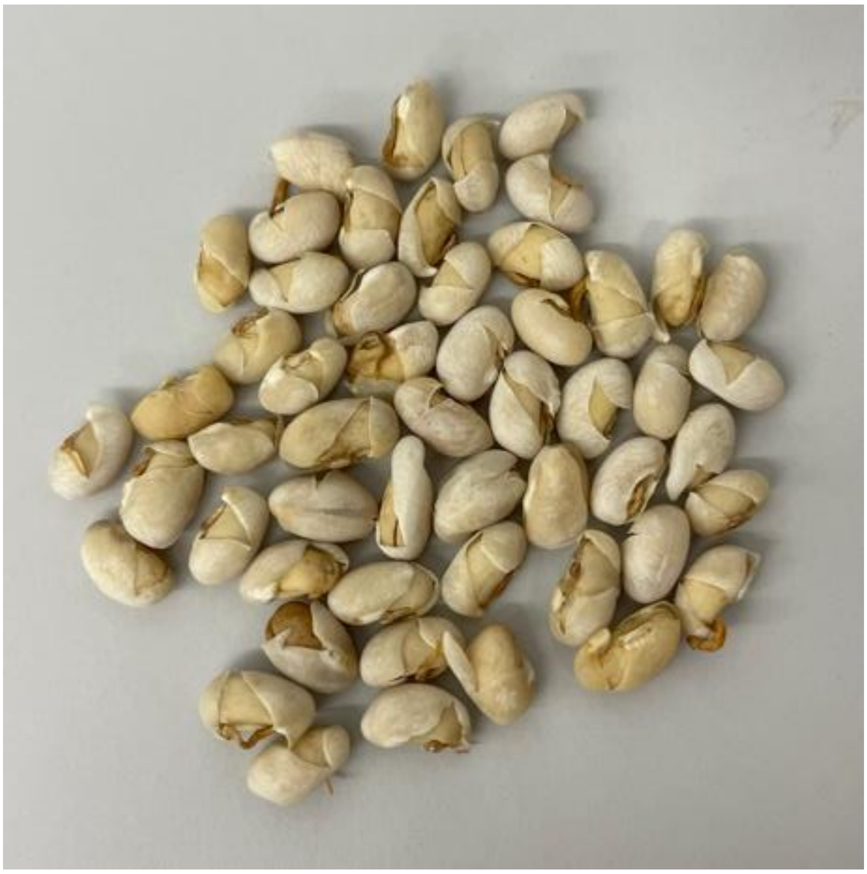

(b)

(c) 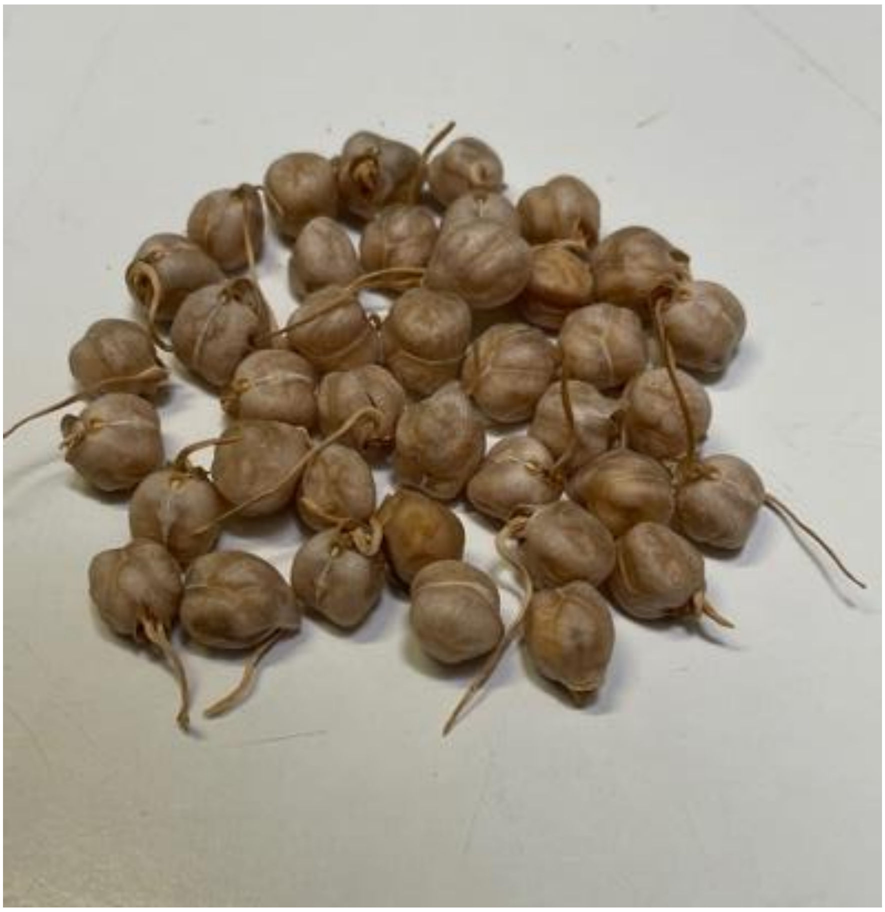

(d) 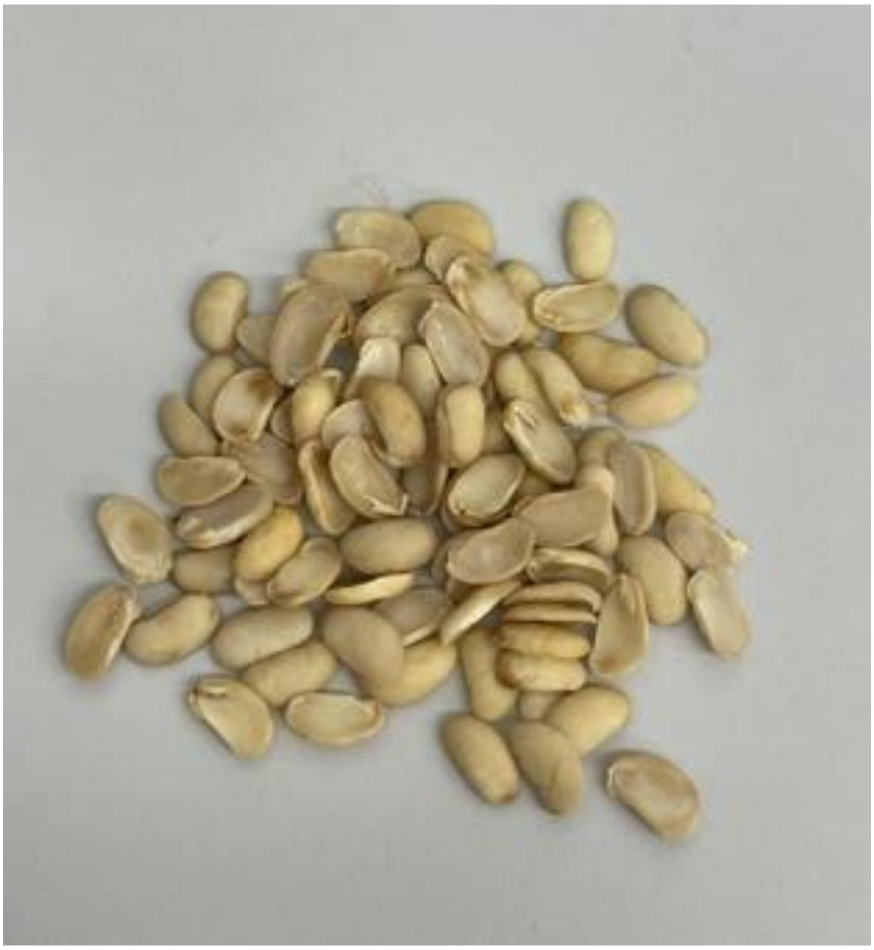

(e) 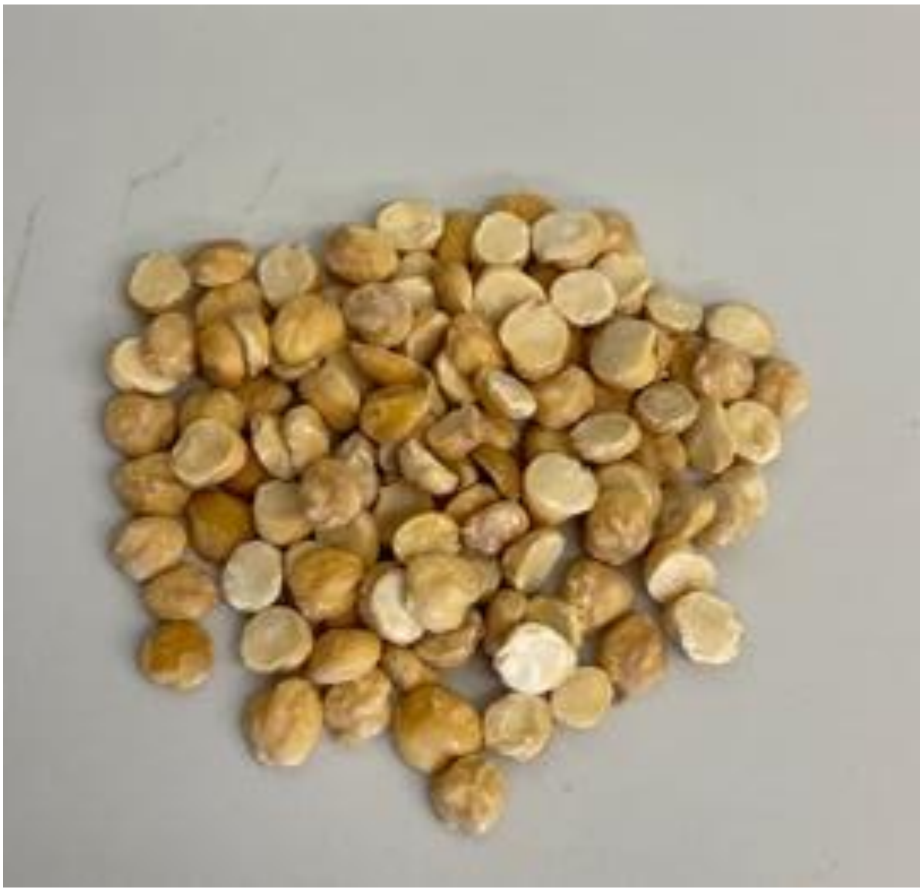

Figure S3

Pictures of the Gradoli Purgatory beans and *Solco Dritto* chickpeas, previously soaked in deionized water at 25 °C for 3 h, when kept germinating in sealed boxes at the same temperature for times ranging from 0 (**a**) to 24 (**b**) or 48 (**c**)

Gradoli Purgatory beans **Solco Dritto chickpeas**

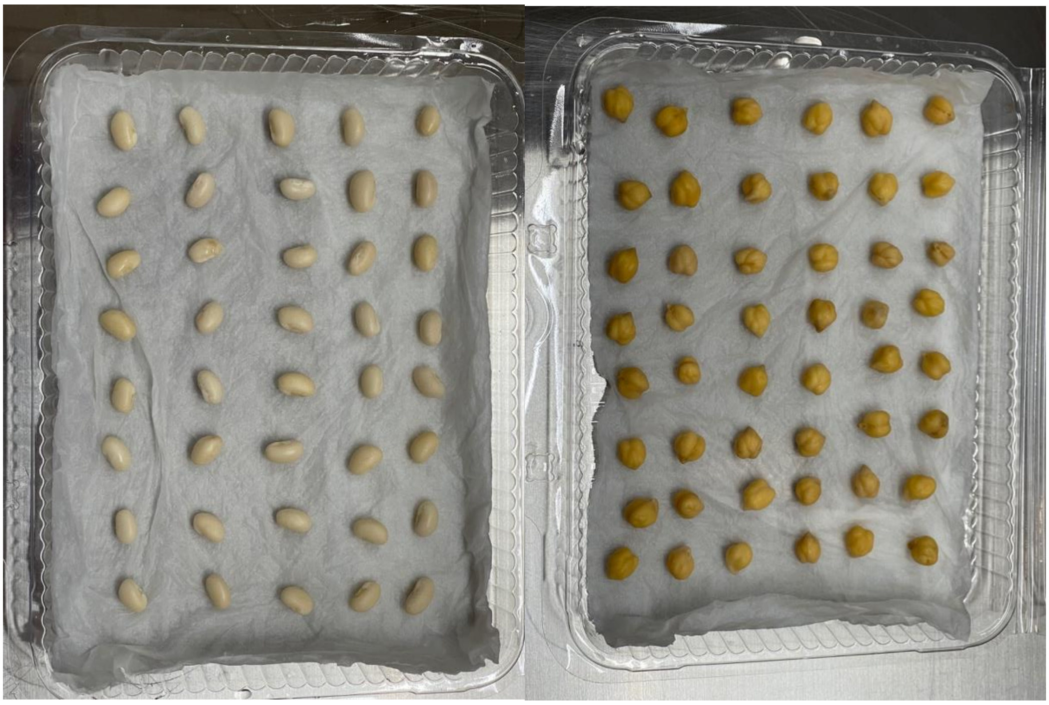

a)

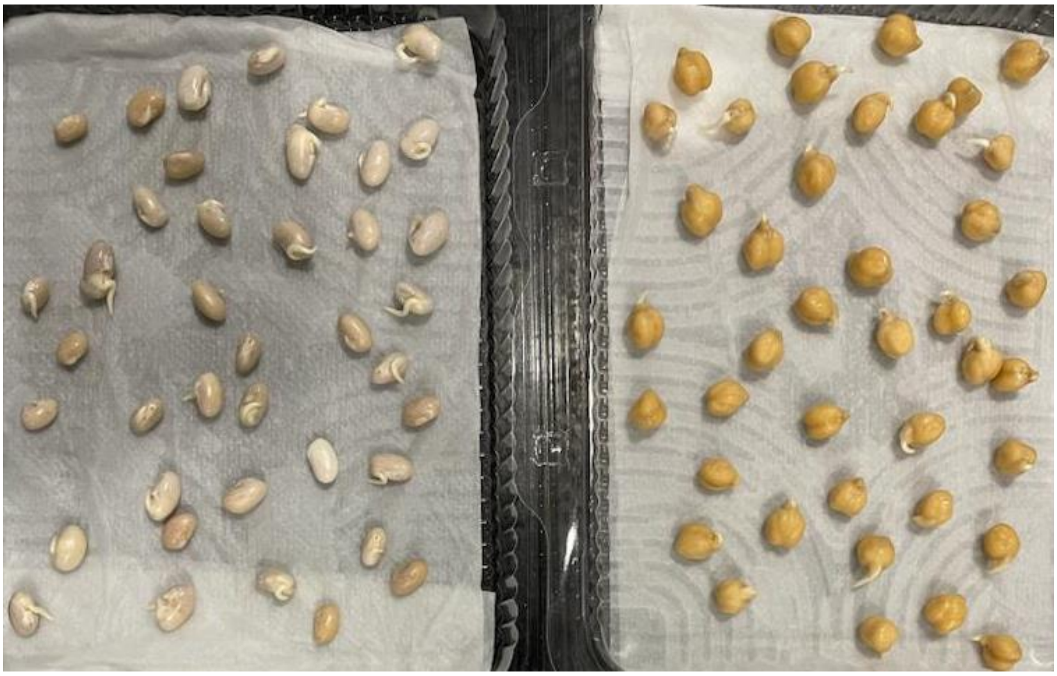
(b)

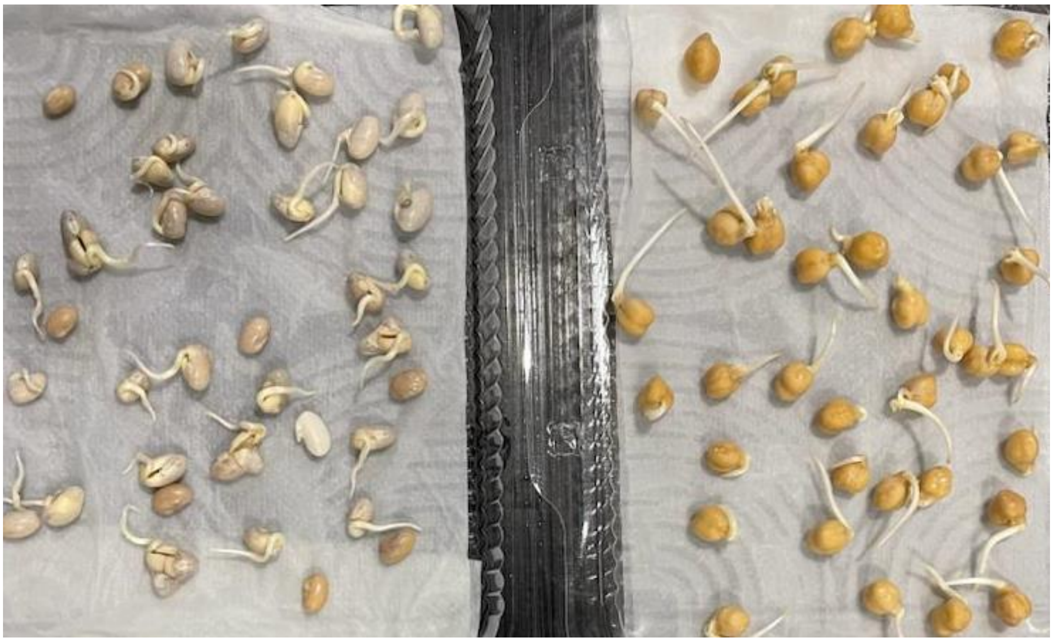
(c)
